# Genetic analysis and preliminary mapping by BSA-seq of the *CmSR* gene regulating the spotted rind trait in melon (*Cucumis melo* L.)

**DOI:** 10.1590/1678-4685-GMB-2024-0062

**Published:** 2024-08-19

**Authors:** Weiyan Zhang, Huijun Zhang, Xiuxiu Zhu, Yahui Li, Guoliang Yuan, Jian Ma

**Affiliations:** 1 Nantong College of Science and Technology, Nantong, Jiangsu, P.R. China; 2 Huaibei Normal University, School of Life Science, Anhui Province Watermelon and Melon Biological Breeding Engineering Research Center, Huaibei, Anhui, P.R. China; 3 Ministry of Agriculture and Rural Affairs, Key Laboratory of Biology and Genetic Improvement of Horticultural Crops (North China), Beijing, P.R. China.; 4 Beijing Academy of Agriculture and Forestry Sciences, Beijing Vegetable Research Center (BVRC), Beijing, P.R. China.

**Keywords:** Melon, rind color, CmSR, spotted rind, molecular marker

## Abstract

Melon (*Cucumis melo* L.) is an economically important horticultural crop. Spotted rind at maturity is an important appearance quality trait in melons. However, the gene controlling this trait remains unknown. In this study, the inheritance pattern of this trait was explored, and the candidate gene underlying this trait was also successfully identified. Genetic analysis showed that a single dominant gene, *Cucumis melo Spotted Rind* (*CmSR),* regulates the spotted rind trait. A preliminary genetic mapping analysis was conducted based on a BSA-seq approach. The *CmAPRR2* gene was identified to be linked with the spotted rind trait and was located on the short arm of chromosome 4. It harbored two single-nucleotide mutations (chr4: 687014 G/A and chr4: 687244 C/A) in the non-spotted line ‘Yellow 2’, which may result in the alternative splicing of the transcript and an amino acid change in the respective protein, from proline to glutamine, respectively. Moreover, marker SNP687014-G/A was developed and co-segregated with the spotted rind trait. Therefore, it is speculated that the *CmAPRR2* gene may be involved in the regulation of the spotted rind trait in melon. This study provides a theoretical foundation for further research on the gene regulatory mechanism of the rind color in melon.

## Introduction

Melon is an annual trailing herbaceous plant of the Cucurbitaceae family, and it is cultivated worldwide (Garcia-Mas et al., 2012). Because of its short cultivation period and high production capacity, melon is a highly economically important crop that can generate high revenues for the farmers and is widely preferred by the consumers. China is the leading country globally in melon planting area and yield production (Wang et al., 2020). According to statistics from FAOSTAT (http://www.fao.org/faostat/en/#data), the melon cultivation area exceeded 354,500 hectares, and more than 12.73 million tons were produced in China in 2018, accounting for around 33.85% and 46.54% of the world’s total cultivation area and yield. There are two types of melon: *C. melo* ssp. *agrestis* (thin-skinned) and *C. melo* ssp. *melo* (muskmelon) (Liu et al., 2020). The thin-skinned melons is characterized by rich aroma, delicious taste, and high sweetness. Meanwhile, it is rich in nutrients such as glucose, malic acid, vitamins, and amino acids, and therefore, it is particularly favored by consumers (Guo et al., 2022).

Rind color is the most direct indicator of the melon fruit appearance quality. Research on rind color has been undertaken on various crops, such as tomato, pepper, cucumber, and bottle gourd (Pan et al., 2013; Liu et al., 2016; Huo et al., 2023). These studies showed that rind color formation is associated with the type and content of pigments in the fruit, which mainly include carotenoids, chlorophyll, anthocyanins, and flavonoids (Tao et al., 2003; Borovsky et al., 2013). The rind color in melon fruits begins to change in the early stage of fruit development, and during the young fruit stage, it is green. As the fruit matures, its rind color undergoes significant changes, exhibiting a green, white, yellow, or orange color, and sometimes various colors mixed and reticulated (Gusmini and Wehner, 2005; Tadmor et al., 2010). An early study suggested that the green rind of melon is dominant relative to white and is genetically controlled by a single gene - *Wi* (Kubicki, 1962). The white rind trait in the ‘Honeydew’ variety is recessive relative to the green rind of the variety ‘Smiths Perfect cantaloupe variety’ (Hughes, 1948). Oren et al. (2019) used the dark green-colored melon accession ‘Dulce’ and light green-colored accession ‘Tam Dew’ to construct segregating populations and perform gene mapping. They identified *CmAPRR2* (*MELO3C003375*) as the key gene that controls rind color (Oren *et al*., 2019). In fruits with light-colored rind, this gene mainly harbors two mutations: one SNP mutation (G→T) in exon 8 and a 13-bp insertion in exon 9. Both mutations lead to premature translation termination of the *CmAPRR2* gene (Oren *et al*., 2019). Feder et al. (2015) used yellow and white rind melon accessions to map the genes that control the yellow rind color trait. As a result, the *CmKFB* gene encoding an F-box protein was identified as the key gene controlling the yellow rind color. This gene is a negative regulator of naringin chalcone accumulation (Feder *et al*., 2015). Similarly, using a genome-wide association study, the *CmKFB* gene was also identified to control the yellow rind color in melon (Gur et al., 2017). Using 635 melon accessions, Zhao et al. (2019) conducted a genome-wide association study to identify genes regulating rind color. They concluded that the green rind color trait was dominant to white and that white was dominant to yellow. Moreover, *CmAPRR2* and *CmKFB* were significantly associated with the green and yellow rind color, respectively, and *MELO3C003097* is a minor-effect regulatory gene (Zhao *et al.*, 2019). In addition, Lv et al. (2018) carried out fine mapping of the *CmSp-1* that controls the striped to non-striped trait of melon, and they located it to a 280.872 kb interval between the markers I734-2 and I757 on chromosome 2. Liu *et al*. (2019) used the green striped melon X010 and the white rind melon M1-113 to construct a genetic population and mapped the *st3* gene, locating it to a 172.8 kb interval between markers M-4-28 and M-4-27 on chromosome 4. Although many studies have been conducted on melon rind traits, the key gene responsible for spotted rind in melon has not yet been identified.

In this study, two thin-skinned melon inbred lines, ‘Spotted 1’ and ‘Yellow 2’, differing in rind appearance, were used for the genetic study of the spotted rind trait. After constructing a genetically segregating population, a bulked-segregant analysis sequencing (BSA-seq) method was employed to locate the *CmSR* gene that controls the spotted rind trait of melon. Our results lay the foundation for revealing the molecular mechanisms underlying the spotted rind formation in melon.

## Material and Methods

### Plant material

The inbred lines ‘Spotted 1’ and ‘Yellow 2’, belonging to the melon subspecies *C. melo* ssp. *agrestis* var. *momordica* were used in this study and were preserved in controlled conditions. F_1_ plants were obtained by crossing ‘Spotted 1’ and ‘Yellow 2’ and self-pollinated to obtain the F_2_ population. All plants were grown in the Wupu Farm in Huaibei City, Anhui Province, with conventional field production management.

### Assessment of fruit rind color

The rind color of individual plants of both parents and F_1_ and F_2_ populations was assessed on fruits 30 days after flowering (DAF). The fruits were visually inspected and photographed (Figure S1).

### BSA-seq analysis

DNA was extracted from 30 spotted rind plants and 30 non-spotted rind plants selected from the F_2_ population using the CTAB method (Murray and Thompson, 1980). The DNA isolated from each plant was mixed in equal amounts to construct two mixed pools, Hua-pool and Huang-pool. Then, two DNA libraries were constructed and submitted to Biomarker Technologies Corporation (Beijing, China) for sequencing analysis. The raw reads were filtered to obtain clean reads. The reads filtering criteria are as follows: (1) the reads with adaptor sequences were trimmed; (2) when the number of bases containing N in the single-end reads was more than 5, the paired end reads were excluded; (3) when the number of bases with low quality (Q <= 15) in single-end sequencing read exceeds 40% of the read length ratio, paired end reads should be removed. After filtering the low-quality raw data, the clean reads from the two pools were aligned to the reference genome with Burrows-Wheeler Aligner (BWA) software (Li and Durbin, 2009). The ‘DHL92’ v3.6.1 (http://cucurbitgenomics.org/organism/18) genome was used as the reference genome. The Euclidean distance (ED) values were used to identify candidate regions related to the spotted rind (Hill et al., 2013) A flowchart of the analysis steps is shown in Figure S2.

### Marker development and validation

A dCAPS marker SNP687014-G/A was designed using the online software dCAPS Finder 2.0 (http://helix.wustl.edu/dcaps/dcaps.html). Then, PCR amplification was performed. The PCR reaction mixture consisted of 25 μl of 2X PrimeSTAR Max Premix (TaRaKa, Beijing), 1 μL of forward primer (10 μM), 1 μL of reverse primer (10 μM), 2 μL of genomic DNA (50 ng/μL), and 21 μL of sterilized distilled water. The PCR cycling conditions were as follows: initial denaturation at 98 ℃ for 2 min, followed by 35 cycles for 10 s at 98 ℃, 5 s at 55 ℃, 30 s at 72 ℃, and finally, extension at 72 ℃ for 5 min. The PCR products were digested immediately with the restriction endonuclease *Bgl*II (Thermo Scientific, USA), including 25 μL PCR products, 3 μl 10×FastDigest^®^ green buffer, 2 μL *Bgl*II at 37 ℃ for 4 hours. The digested products were detected by 8% polyacrylamide gel electrophoresis. The primer used for this study is listed in Table 1.

**Table 1 -:** Primer sequences used in this study.

Primer name	Forward primer (F, 5’-3’)	Reverse primer (R, 5’-3’)	Endonuclease	Expected size
SNP687014-G/A	CCCTATCATGTGTGTGTTCTCC	TCACAATTTTTGCTAGAACTGAACAA*G*	*Bgl* II	198/174 bp

## Results

### Phenotypic and genetic analyses of the spotted rind

**Figure 1 -:**
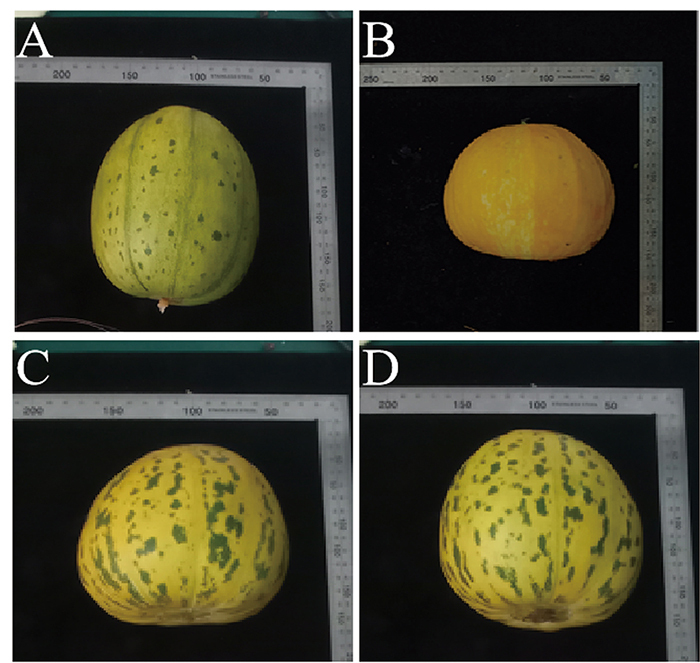
Rind colors of parental and reciprocal cross F_1_ plants. (A), Fruit phenotype of the parent ‘Spotted 1’; (B), Fruit phenotype of the parent ‘Yellow 2’; (C), F_1_ fruit phenotype (‘Spotted 1’ as the female parent, ‘Yellow 2’ as the male parent); (D), F_1_’ fruit phenotype (‘Spotted 1’ as the male parent, ‘Yellow 2’ as the female parent).

By observing the rind color of mature fruits (30 DAF), it was found that the parent ‘Spotted 1’ displayed a yellow-spotted rind (Figure 1 A ). In contrast, the parent ‘Yellow 2’ displayed a plain yellow, non-spotted rind (Figure 1 B ). Reciprocal crosses were performed using the ‘Spotted 1’ and ‘Yellow 2’ parents to explore the genetics underlying the spotted rind phenotype. The rind color of the obtained F_1_ generation was the same as the parent ‘Spotted 1’, exhibiting a yellow spotted rind. This suggests that the spotted rind phenotype is controlled by dominant nuclear genes, which are dominant to the plain yellow non-spotted rind phenotype (Figure 1 C , D). Subsequently, a phenotypic observation was conducted on the rind color of 170 F_2_ lines. The results showed that 124 F_2_ lines carried fruits with spotted rind and 46 F_2_ lines with non-spotted rind, fitting a 3:1 ratio of Mendelian segregation (χ^2^_0.05_ = 0.38 <3.84). These results indicate that the spotted rind is a qualitative trait controlled by a single gene, temporarily termed *CmSR* (*Cucumis melo Spotted Rind*).

### Preliminary localization of the *CmSR* gene

**Figure 2 -:**
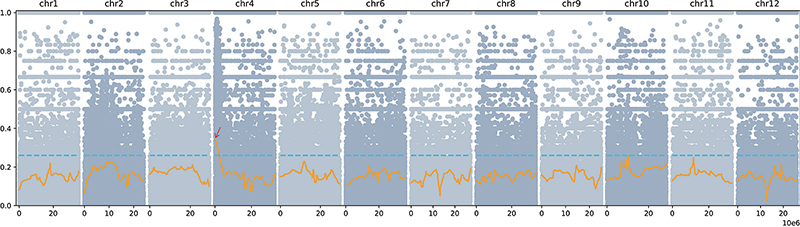
Manhattan plot of the *CmSR* gene based on ED algorithm. Each coloured dot represents an ED-based linkage value of an SNP site. The orange lines represent the fitted ED value. The blue dotted line represents the threshold for the screening of the candidate region. The window larger than the threshold at 99% confidence level was selected as the candidate interval indicating with the red arrow.

To primarily determine the chromosome location of the *CmSR* gene that controls the spotted rind phenotype, BSA-seq based on genomic DNA re-sequencing was conducted. First, 30 F_2_ plants with spotted rind and 30 F_2_ plants with non-spotted rind were selected from the F_2_ population. The DNA of the individual plants was extracted, and the spotted Hua-pool and non-spotted line Huang-pool were established. Subsequently, the constructed bulked DNA pools were used for genome re-sequencing using Illumina HiSeq^TM^ 2500, with a sequencing depth of 30X. An average of total 15.48 Gb filtered clean reads were obtained, with an average of 96.82% reads at a Q20 value, and were mapped to the reference DHL92 v3.6.1 reference genome (Castanera et al., 2020; http://cucurbitgenomics.org/organism/18). High-quality SNPs were filtered through the GATK software (V3.7) and used for association analysis according to the Euclidean distance (ED) algorithm by DeepBSA software (McKenna et al., 2010; Hill et al., 2013; Li et al., 2022). A confidence interval (99%) significantly associated with the spotted rind trait was identified at the top end of chromosome 4, located within the 0.007~3.57 Mb region, spanning 3.57 Mb (Figure 2). The preliminary BSA-seq mapping results suggested that the *CmSR* gene might be located in this candidate genomic region.

### Analysis of candidate genes within the mapped interval

According to previous reports, the *MELO3C003375* gene (i.e., *CmAPRR2*), located on the short arm of chromosome 4, is related to the rind color of melon. Therefore, its gene sequences in the parents ‘Spotted 1’ and ‘Yellow 2’ were initially examined. Gene re-sequencing results showed that the *MELO3C003375* gene carried a G to A mutation and a C to A mutation on chr4: 687014 and chr4: 687244, respectively, in ‘Yellow 2’. The former is located at the last base of the fifth exon and might have caused alternative splicing of mRNA, with the latter leading to an amino acid residue change from proline (Pro) to glutamine (Gln). From this, it was speculated that *MELO3C003375* (*CmAPRR2*) is likely the target candidate gene controlling the spotted rind phenotype in melon.

### Development and validation of molecular marker

**Figure 3 -:**
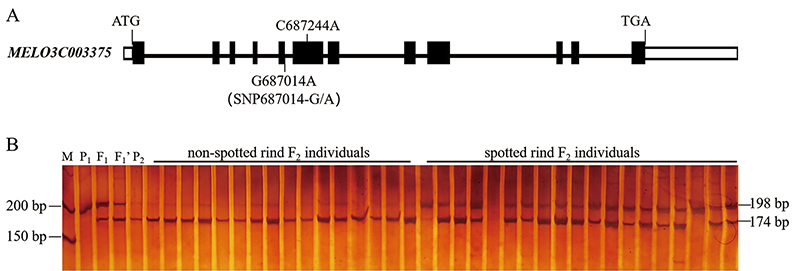
The gene structure of candidate gene and development of molecular marker. (A), The sequence variations and gene structure of *MELO3C003375*. UTR, exons and introns are indicated by white rectangles, black rectangles and black lines, respectively. (B),Validation of SNP687014-G/A marker on parents, F_1_, F_1_’, and partial F_2_-generation individuals. M: DL500 DNA marker; P_1_: parent ‘Spotted 1’; P_2_: parent ‘Yellow 2’; F_1_: ‘Spotted 1’^♀^× ‘Yellow 2’^♂^); F_1_’: ‘Spotted 1’^♂^×‘Yellow 2’^♀^.

In order to clarify the relationship between the SNP variation in the *CmAPRR2* gene and the spotted rind phenotype, a dCAPS maker SNP687014-G/A was developed based on the G to A mutation on the chr4: 687014 site using the online software dCAPS Finder 2.0. A mismatched base, G, was introduced at the end of the reverse primer to form a specific cleavage site for the restriction endonuclease *Bgl*II. Then, 170 F_2_ individuals were genotyped by the marker SNP687014-G/A. The results showed that marker SNP687014-G/A co-segregated with the spotted rind trait (Figure 3), indicating that the variation in the *CmAPRR2* gene was associated with the spotted rind trait.

## Discussion

Rind color is an important agronomic trait of melon, and its genetic control has been a focus of research. Previous studies have demonstrated that the *CmAPRR2* gene located on chromosome 4 and the *CmKFB* gene on chromosome 10 of the melon genome are the two main functional genes that control the green and yellow color of melon rind (Oren et al., 2019; Zhao et al., 2019). Among them, the *CmAPRR2* gene encodes a Golden2-like transcription factor and this gene family has been reported to regulate plastid development and chlorophyll metabolism of the fruit rind in cucumber, tomato, and other crops (Pan et al., 2013; Liu et al., 2016; Ma et al., 2021; Zhu et al., 2022; Fang et al., 2023). In this study, genetic analysis indicated that the spotted rind phenotype of melon is controlled by a single dominant gene, *CmSR*. Using the BSA-seq method, the gene was located on the short arm of chromosome 4, with 2 single base mutations present in the *CmAPRR2* allele within the interval*.* The mutations may lead to alternative splicing and amino acid variation in the transcript and protein, respectively.

*CmAPRR2* is homologous to the *Arabidopsis pseudo-response regulator 2* (*APRR2*) gene, a specific plant transcription factor. It is derived from authentic response regulators (ARRs) and belongs to the pseudo-response regulator (PRR) family (Hosoda et al., 2002). A typical PRR2 protein contains a receiver-like domain (RLD) and a Golden-2-like (GLK) motif containing the Myb-like DNA binding domain (Chen et al., 2016). GLK transcription factors belong to the GARP superfamily, which plays key regulatory roles for the expression of photosynthesis-related genes in leaves and fruits and the development of chloroplasts (Riechmann et al., 2000; Hosoda *et al.*, 2002; Waters et al., 2008; Nguyen et al., 2014). These genes were thought to be associated with increased plastid number and chlorophyll accumulation in immature fruits, resulting in white or light green rind. Studies have shown that *APRR2* and its homologous genes can regulate chlorophyll content and thus affect rind color in many Solanaceae and Cucurbitaceae vegetables, such as tomato, pepper, cucumber, bitter melon, watermelon, and wax guard (Pan et al., 2013; Oren et al., 2019; Arrones et al., 2022; Tian et al., 2023). In cucumber, *APRR2* controls the rind color in immature fruits (Liu et al., 2016). The premature termination of the protein encoded by this gene causes a change in the young fruit peel color from green to white (Liu *et al*., 2016). In melon, Xu et al. (2021) showed that in the light green parent, ‘LGR’, a G to T mutation is present in the coding region of the *CmAPRR2* gene, resulting in premature termination of protein translation with most of its Myb-DNA binding domain being truncated. Therefore, it has been proposed that *CmAPRR2* is the major candidate gene affecting the rind color of immature melon fruits (Xu *et al*., 2021). In this study, the rind of the immature fruits of the parent ‘Spotted 1’ was green, and when they matured, they exhibited a yellow rind with green spots. It is speculated that it is caused by incomplete chlorophyll degradation during fruit ripening. It is possibly also related to the metabolism of chlorophyll, as suggested by the function of the candidate gene *CmAPRR2*.

Rind color is an important quality trait of melon and an important objective of melon breeding. The ‘Hualei’ type melons are oriental melon types with superior quality and high yield. The fruit rind of ‘Hualei’ is spotted, which is favored by consumers, but the mechanism regulating the spotted rind is unknown. Through genetic analysis and gene mapping, this study preliminarily clarified that the *CmAPRR2* gene is the key gene controlling the spotted rind trait, and a dCAPS marker SNP687014-G/A was developed and co-segregated with the spotted rind trait. This study lays the foundation for future molecular breeding programs on melon rind color and may aid the elucidation of the detailed molecular mechanisms underlying the *CmAPRR2* regulation of the spotted rind phenotype in melon.

## Conclusions

In this study, the inheritance pattern of the spotted rind trait was explored in melon. Genetic analysis revealed that a single dominant gene *CmSR* controls spotted rind of melon. A candidate interval located on the short arm of chromosome 4 was associated with the spotted and non-spotted rind traits based BSA-seq. And a candidate gene (*MELO3C003375*) was mapped to this interval, which was predicted to encode an APRR2 protein regulated the rind color in previous studies. Subsequently, gene re-sequence analysis revealed that two SNP mutations in the non-spotted line ‘Yellow 2’ led to alternative splicing and an amino acid change in the predicted protein. Genotypic validation of 170 F_2_ individuals using the dCAPS marker SNP687014-G/A co-segregated with *CmAPRR2* gene could predict the rind color (spotted/non-spotted) trait with an accuracy of 100%. This study will be valuable for molecular marker-assisted selection in melon breeding and provides a theoretical foundation for further research on the formation mechanism of the rind color in melon.

## Supplementary material

The following online material is available for this article:

Figure S1 - The images of partial F2 individuals.

Figure S2 - Flowchart of information analysis procedure.
