# Supplementary material for: Genetic analysis and preliminary mapping by BSA-seq of the CmSR gene regulating the spotted rind trait in melon (Cucumis melo L.)
Source: Genet Mol Biol. 2024 Aug 19;47(3):e20240062. doi: 10.1590/1678-4685-GMB-2024-0062 (PMC11334433; doi:10.1590/1678-4685-GMB-2024-0062)
Supplement: Figure S1 - [file 1415-4757-GMB-47-03-e20240062-s1.pdf]

Genetics and Molecular Biology
https://doi.org/10.1590/1678-4685-GMB-2024-0062

Supplementary Material to "Genetic analysis and preliminary mapping by BSA-seq of the CmSR gene regulating the spotted rind trait in melon (*Cucumis melo* L.)"

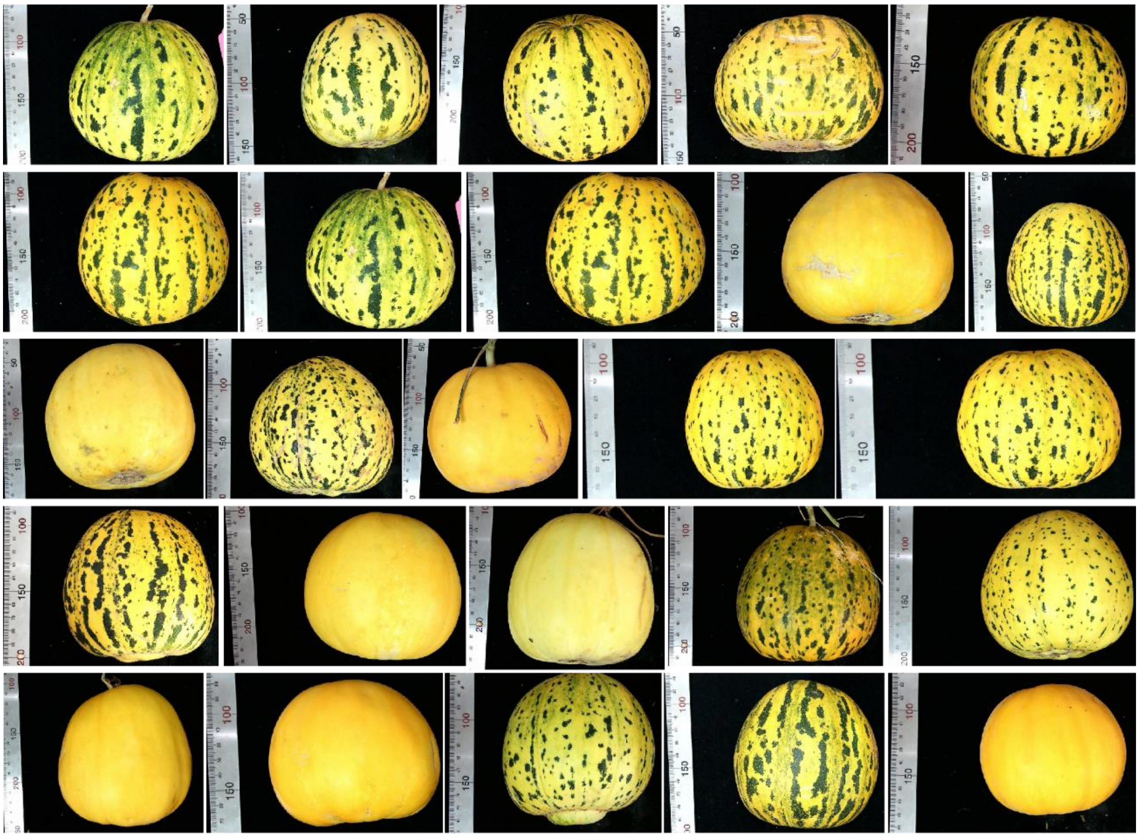

Figure S1 - The images of partial F_2 individuals.
